# Tanzanian farmers' knowledge and attitudes to GM biotechnology and the potential use of GM crops to provide improved levels of food security. A Qualitative Study

**DOI:** 10.1186/1471-2458-10-407

**Published:** 2010-07-12

**Authors:** Christopher P Lewis, James N Newell, Caroline M Herron, Haidari Nawabu

**Affiliations:** 1 School of Medicine, University of Leeds, UK, LS2 9JT; 2 The Nuffield Centre for International Health & Development Leeds Institute of Health Sciences, University of Leeds, Charles Thackrah Building, 101 Clarendon Road, Leeds, UK, LS2 9LJ; 3 Formerly of The International Institute of Tropical Agriculture (IITA), Plot 331, Kambarage Road Mikocheni A, P.O. Box 34441, Dar-es-Salaam, Tanzania; 4 The International Institute of Tropical Agriculture (IITA), Plot 331, Kambarage Road Mikocheni A, P.O. Box 34441, Dar-es-Salaam, Tanzania

## Abstract

**Background:**

Genetically Modified (GM) crops have been championed as one possible method to improve food security and individual nutritional status in sub Saharan Africa. Understanding and acceptability of GM crop technology to farmers and consumers have not been assessed. We developed a qualitative research study involving farmers as both producers and consumers to gauge the understanding of GM crop technology, its acceptability, and identifying issues of concern.

**Methods:**

Nineteen individual interviews (10 male and 9 female) and five mixed gender focus group discussions with local farmers were conducted in 3 regions in Tanzania. Analysis took place concurrently with data collection. Following initial interviews, subsequent questions were adjusted based on emerging themes.

**Results:**

Understanding, awareness and knowledge of GM crop technology and terminology and its potential risks and benefits was very poor in all regions. Receptivity to the potential use of GM crops was, however, high. Respondents focused on the potential benefits of GM crops rather than any potential longer term health risks. A number of factors, most significantly field trial data, would influence farmers' decisions regarding the introduction of GM crop varieties into their farming practice. Understanding of the potential improved health provision possible by changes in agricultural practice and food-related decision making, and the health benefits of a diet containing essential vitamins, minerals and micronutrients is also poor in these communities.

**Conclusion:**

This study forms a basis from which further research work can be undertaken. It is important to continue to assess opinions and attitudes of farmers and consumers in sub Saharan Africa towards potential use of GM technologies whilst highlighting the importance of the relationship between agriculture, health and development. This will allow people in the region to make accurate, informed decisions about whether they believe use of GM biotechnology is an appropriate way in which to tackle issues of food security, provide improved health and drive development.

## Background

Good nutrition forms a foundation for human health and development, and the link between poor nutrition and poor health has long been established [1]. Food security, "when all people at all times have access to sufficient, safe and nutritious food to maintain a healthy and active life" [2], represents a major cause for concern in sub-Saharan Africa. Poor nutrition is a significant contributory factor to poor health, especially in children [3]. Over recent years, although food productivity has improved and the total number of people who are undernourished has fallen, the nutritional balance of diets remains very poor [1]: increased numbers of people, particularly children under five in sub-Saharan Africa, suffer from health problems associated with vitamin A, zinc and iodine deficiencies. All these conditions can be prevented by consumption of an adequately varied diet with a balanced vitamin and micronutrient content [3]. It is therefore important that strategies are found to provide not only improved basic food security but also improved overall nutritional quality of diets. The use of Genetically Modified (GM) crops has been championed as one method of improving food security and nutritional status in low-income countries by increasing food productivity [4]. However, there is also considerable opposition to the use of GM crops. Table 1 gives some potential benefits and risks of GM crops. Although there have been a number of successes with regard to the development of GM biotechnologies, there is a lack of appropriate biosafety regulations, protocols and stewardship schemes in many developing countries [5].

**Table 1:** Some potential benefits and issues of concern regarding the use of GM crops (Sources; References [4,6,10])

Potential Benefits	Potential Issues of Concern
Improved resistance to pests, disease and herbicides; for instance, destruction by common agricultural pests and diseases, such as nematodes, insects, fungal, bacterial viruses, or parasitic weeds.	Potential human health impacts; for instance, allergens, transfer of antibiotic resistance, and unknown effects

Improved yields, taste, quality or nutritional value; for instance biofortification with essential vitamins (eg A, C or K) or minerals (eg folic acid or beta carotene).	Potential environmental impacts; for instance, unknown effects on other organisms, unintended transfer of transgenes through cross-pollination and the loss of flora and fauna biodiversity

Improved tolerance to environmental stresses including prolonged drought, high salinity, increased rainfall or increased temperatures.	Potential loss of access and intellectual property; for instance, the foreign exploitation of natural resources, the dependence of a developing country on a developed country, or the dominance of world food production by one or a few multinational companies

Improved tolerance of reduced growing seasons so that crops need a shorter growing season while providing the same level of production.	Ethical issues, such as tampering with nature by mixing genes between species, objections to consuming animal genes in plants and vice versa, or violating natual organisms' intrinsic values

	labelling issues; mixing non-GM with GM crops may compromise seed or food

In sub Saharan Africa, where 204 million people are estimated to suffer from chronic undernourishment, the majority of daily calories are provided by only a few basic foodstuffs: principally cassava, plantain and maize [6]. This narrow range leaves populations vulnerable to crop failure, reduced crop yields and quality losses due to diseases and pests, losses during storage and the impact of climate change [7,8]. Introduction of GM crops is a potential solution to some of these issues. However, we were concerned that GM crops were being developed for use in Africa without input of any kind from African farmers and consumers: a literature search for studies assessing African farmers' understanding and acceptability of GM crop technology produced only one, not widely accessible, study [9]. We therefore planned research in Tanzania to help fill this gap. A quantitative survey was inappropriate as the issues related to the widespread use of GM crops from the viewpoint of village communities are not well enough understood by local communities. We therefore developed a qualitative research study involving farming families as both food producers and consumers, with the objectives of identifying some of the issues related to local understanding of GM crop technology and its acceptability, and identifying concerns, if any, of farming communities that would need to be addressed prior to considering any trials of newly developed GM crop varieties designed to improve food security, nutrition and health. A research project was underway to develop GM cassava resistant to cassava brown streak disease and cassava mosaic disease (both caused by virus pathogens), at Mikocheni Agricultural Research Institute, (MARI), in Tanzania, in collaboration with the International Institute for Tropical Agriculture (IITA). We therefore chose transgenic cassava as a specific example to lead and generate discussion with farmers on the understanding and acceptability of GM crops. We hope the findings will be informative and add to the debate about how best to engage local farming communities in decisions about the use or otherwise of GM crops in Tanzania in their farming practice. Such community participation is recommended by a recently developed Tanzanian legal biosafety framework, which requires any research involving GM crops to follow regulatory guidelines [11] to minimize the potential for human or animal harm.

## Methods

**Figure 1:**
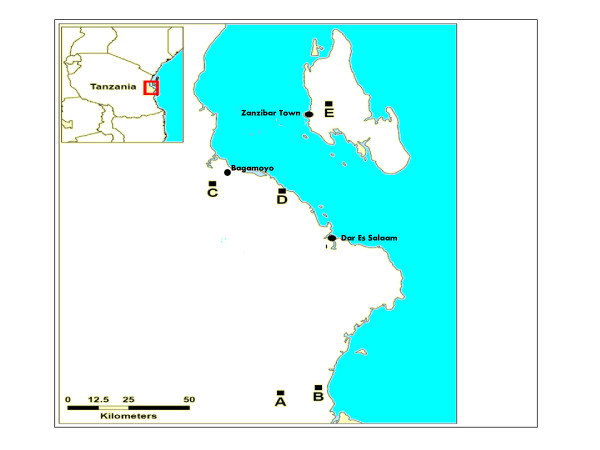
**Focus group discussion sites**.

During June 2009, in-depth interviews and focus group discussions (FGDs) were conducted in five sites in three administrative districts near Dar es Salaam, Tanzania (Figure 1). These sites were purposefully chosen to represent different geographic regions and local demographics (Table 2). In three of the sites (B, C, D) subsistence farming was practised, with crop yields being consumed entirely by local farming communities themselves. In the two other sites (A, E), there were sometimes surpluses, which were then sold at local markets. Site A is actively involved in the trialling of new varieties of farming crops produced by MARI and IITA using conventional plant breeding techniques.

**Table 2:** Location and demographic details of the five sites visited

Site	Village Name	Main Type of Farming Practice	Number of Households	Population	Nearest Urban Centre (distance/km)
	(District)				
A	Sululu-Bungu	Mainly subsistence, small surpluses in some years	600	1791	Dar es Salaam (100 km)
	**(Rufiji)**				

B	Nyamangwa-Bungu	Subsistence	353	1783	Dar es Salaam (105 km)
	**(Rufiji)**				

C	Yombo-Yombo	Subsistence	120	420	Bagamoyo (8 km)
	**(Bagamoyo)**				

D	Matimbwa-Matimbwa	Subsistence	75	360	Bagamoyo [12]
	**(Bagamoyo)**				

E	Machui-Unguja	Mainly subsistence, small surpluses in some years	860	2250	Zanzibar Town (30)
	**(Zanzibar)**				

In total, 19 in-depth individual interviews (11 male and 9 female) were conducted. Following these initial interviews, we carried out five mixed gender FGDs, one at each site, each with 7 local farmers (Table 3). These took place at the communal village centre areas at each of the five sites. Both individual interviews and FGDs were based around semi-structured questions (appendix). Individual interviews were conducted to gain insight into individual understanding of GM crop technology and its acceptability to the individual farmers. They were also designed to give opportunities for farmers to discuss the wider aspects of individual farming practices and traditions, and to raise any issues related to on their agricultural practice and their families' food security. FGDs were then conducted to further explore themes emerging from individual interviews. At each site, after permission was given by each village leader and local Agricultural Extension Officer (AEO), individuals from each farming community were asked to volunteer for both individual interviews and FGDs. (The AEO is a local district council employee trained by the Ministry of Agriculture, Food and Cooperatives, (MAFC), who is responsible for overseeing agricultural and livestock farming practices.) In addition, separate interviews were conducted with each of the 3 regional AEOs.

**Table 3:** Numbers of participants' individual interviews and focus group discussions at each of the five sites

Site	Numbers of individual interviews *(male/female)*	Numbers of participants in each focus group discussion *(male/female*	Agricultural Extension officer interviewed *(sex)*
A	4 *(2/2)*	7 (5/2)	AEO for Rufiji *(male)*

B	4 *(2/2)*	7 (3/4)	AEO for Rufiji *(male*)

C	4 *(2/2)*	7 (6/1)	AEO for Bagamoyo *(male)*

D	4 *(2/2)*	7 (3/4)	AEO for Bagamoyo *(male)*

E	3 *(2/1)*	7 (5/2)	AEO for Zanzibar (*male)*

Following initial interviews, subsequent questions were adjusted based on emerging themes. During interviews and FGDs the interviewer/facilitator took notes and made audio recordings which were then transcribed and coded based on constant comparison of emergent themes, ideas, concepts, phrases and keywords. All respondents gave written informed consent before each interview and FGD. Research permission, including ethical committee approval was granted by the Tanzania Commission for Science and Technology, COSTECH, Tanzania.

Prior to the interviews and FGDs, and in consultation with health and plant scientists, we developed an explanatory statement designed to provide an appropriate, informative and unbiased overview of GM biotechnology in both Kiswahili and English (see appendix). We planned to use the statement to facilitate further discussion in cases where a respondent had very limited understanding of GM biotechnology; discussion would then be focused around initial impressions, understanding and issues which arose following this description of GM crop systems.

## Results

### 1. Understanding, knowledge and awareness of GM crop technology and terminology

Understanding, awareness and knowledge of GM crop technology and terminology was very poor in all areas visited. Only three respondents had heard the term "genetically modified" before, all on the radio: all reported that they had no understanding of GM crop technology. Only one attempted an explanation of GM crop technology:

"I think that GM crop technology is like creating a hybrid: you take two different forms of the crop and breed them together to make a better version." (Respondent 2 Site B)

The explanatory statement was therefore necessary in all interviews and FGDs. Following delivery of the explanatory statement, all interviewees and FGDs were asked if they had any questions regarding the processes involved in genetic modification. Four questions arose regularly in all sites:

Are these types of crops like a hybrid?

Will these new crops look the same?

Will these new crops taste the same?

Will these new crops grow in the same way as our current variety?

The three AEOs interviewed also showed very poor understanding and awareness of GM biotechnologies. Two felt that they could accurately describe GM crop technology, but when asked to do so, described it incorrectly as a process of cross fertilization between two already established crop varieties to produce a new variety.

### 2. Receptivity to the potential use of GM crops in farming practice

In general, acceptability and receptivity towards the potential use of GM crops as a possible method to improve food security was high.

" We would be interested in new crop types which might grow better and mean we have more food for our family" (Respondent 4 Site D)

The majority of respondents focused on the potential benefits of GM rather than any potential health risks to either themselves or the local ecosystem.

"What I worry about is making sure I have enough food this year for me and my family. I don't think about long into the future, we don't live like that here". (Respondent 3, Site E)

When asked whether they would consider a trial of GM crops, receptivity depended on many factors including provision of information, previous exposure to farming initiatives, type of farming practice, involvement of scientists in the trial process and provision of incentives (see Table 4).

**Table 4:** Factors affecting farmers' preparedness to be involved in trials of GM crops

Provision of information	Before any trial, farmers would want a chance to speak with those who developed the crops to enable them to learn more and to ask questions regarding GM crop production
	
	All respondents believed they would be given enough information to make an informed, autonomous decision before any trial was undertaken, regardless of the body or organisation conducting the trial.
Previous exposure	Respondents were more receptive to becoming involved in a trial when they had had previous contact with scientists or developmental organisations across a number of disciplines, not solely agriculture.
	
	Where respondents had had little or no contact with such initiatives, their receptivity to trialling GM crop varieties was markedly reduced.

Type of farming practice	All the farmers said that undertaking a trial using a new crop variety would mean sacrificing some land under current cultivation. All the farmers said that they currently farmed the maximum acreage possible given the labour available.
	Where farmers were undertaking subsistence farming, producing just enough crops to provide adequate food, they were more reluctant to take the risk of sacrificing land to test a new crop variety because of the potential consequences of reduced yield if the trial was unsuccessful.
	Those farmers who produced enough crops to allow surplus to be traded felt that potential benefits of testing a new GM crop variety outweighed the potential risk of reduced overall crop production.

Involvement of scientists in the trial process	In all cases farmers preferred that scientists should be involved in all stages of a trial, from planting to harvesting, processing and tasting.

Incentives	All farmers would trial a new GM crop variety if they were paid: their concern about land sacrifice associated with a trial would be countered by financial incentives.
	Respondents would also be less concerned about close involvement of scientists in the trial process if given financial incentives.
	Where farmers had excess land which was fallow, they would have no concerns in allowing scientists to cultivate their spare land in order to test a new GM crop variety in exchange for the final crop products.

Respondents felt that the most important quality which could be given to new GM crop varieties was resistance to disease and pests leading to increased crop yield. Farmers who in good growing seasons sold surplus crops thought that yield and crop taste were equally important.

### 3. Agriculture, nutrition and health

During all interviews, the importance of agriculture in providing good health was discussed. All FGD respondents shared the opinion that it was important to eat a healthy diet, which they considered to be one which provided enough calories to prevent hunger. They agreed that as a community, they did not usually go hungry. They felt their diet did not contain all the types of food they would ideally like: but this concern was secondary to making sure that they didn't go hungry. They felt that, due to lack of availability and high cost, their diet lacked dairy products and animal proteins, but these concerns arose from issues of taste and satiety rather than health provision. Where farmers had surplus crop to be sold, the purchase of non-food items took precedence over supplementing their diet. Income from these sales was spent on essential non-food items such as clothing and transport, and also to a significant extent on cigarettes, alcohol and other non-essential items.

AEOs showed poor understanding of the benefits of balanced nutritional intake to overall health, and of the link between agricultural practice, health and development. All AEOs believed that as long as each farmer was producing enough of any crop so that his family did not go hungry, improvements in their health would be better brought about by other initiatives rather than changes in agricultural practice. Two of the AEOs stated that satisfying daily energy requirements was as much as agricultural practice could contribute to health provision. As a result, they felt it would be very difficult to promote the use of GM crop varieties which were vitamin and nutrient fortified if these crops did not also provide another desirable quality such as increased yield, resistance to disease or drought, or a shorter growing season.

All respondents felt that development of GM crop varieties would be one way to improve both their food security and their overall health. However, they felt that there were other ways they could be helped to increase their crop production, and better initiatives which could be implemented to improve their health. The most commonly mentioned agricultural intervention was provision of better technology to reduce the amount of labour required to farm their land. Regarding health improvement, respondents focused on provision of better medicines and increased access to medical services.

### 4. Factors influencing farmers' potential use of GM crop technology

In general, receptivity to potential use of GM crops as a possible method to improve food security was high. The majority of respondents focused on the potential benefits of GM rather than any potential health risks. All respondents thought that the main influence on their decision to incorporate GM crops into their everyday farming practice would be the result of a field trial. Comparison of end yield was the most significant factor by which they would judge the suitability of any new GM crops. Importance was also placed on comparison of the patterns of growth observed between the two varieties, evidence of any disease and the amount of labour required to produce the crop. Farmers who produced excess in good years would judge success of any new variety using a combination of yield and taste: taste is an important factor in determining market value.

"... how much crop we get is of course very important, but if we take cassava to market, and it has a bitter taste, we will have trouble selling it. It's better to have less crop which is nice [to taste] then lots of crop which is bad [to taste]". (Respondent 6, Site C)

All but one farmer said they would judge the value of a new crop variety after only a single growing season. They felt that because the old and new varieties were subject to the same conditions during their growing period, regardless of how the conditions impacted on crop production, they would we able to make an accurate comparison of the GM and conventional crop varieties.

Farmers who had a small crop surplus reported that they would significantly increase the amount of land given to a new variety after one season of good results because the potential benefits of increased production and income gained would outweigh the risk that the crop might not perform well in its second year. Farmers who produced only enough food to feed themselves and their family were much more wary of quickly increasing the total percentage of their land given to a new crop variety because of the potential loss of overall crop amounts if the new variety performed poorly.

A majority of those interviewed said that the AEO was a major source of influence on their farming decisions. Many said the opinion of the AEO would be very influential in any decision they would make about involvement in trials or use of GM crop varieties. They felt that the AEO was very knowledgeable in farming practices and would have a better understanding of GM crop technology than themselves. They also reported a high level of trust in their AEO because he was a direct employee of the government-elected local council and therefore shared the government's aims of improving their agricultural practice and overall quality of life.

Farmers said that if religious leaders were to advise against GM crop use on religious grounds, they would take this into serious consideration. They said, however, that religious leaders rarely expressed views on agricultural practices. Three respondents gave the example of their Imam's negative comments on the use of a medication for elephantiasis, which made some individuals avoid using it, even though they believed its curative effects were clear in those that used it.

Farmers reported that although debate regarding crop choice and methods of farming practice did occur within families, farming decisions within a family group were always made by the (male) head of the family. Most respondents reported that if they were told about the benefits or failings of a new crop variety or technological development by farmers in the surrounding communities, they would take this into serious consideration when make a decision about its use.

### 5. Issues of concern raised by respondents

Only one respondent raised concern regarding the safety of eating GM crop products;

"... because of the way these crops are made, I would be worried about eating them ... but not if the scientists that were involved in the trial they themselves ate the crops with me ... this would show me they were safe." (Respondent 3, Site A)

All others said that they were not worried about the safety of eating GM crops because they expected that the government would oversee the development and use of these new GM crops. They felt that any possible health risks were greatly outweighed by the potential of these crops to provide them with an increased amount of food for their consumption, improving their short term food security as a result.

Where previous contact with scientists involved in development initiatives had occurred (and not only in agricultural development programmes), respondents reported a high degree of trust and confidence in members of the scientific community. This was particularly apparent at Sites A and E where farming communities had been in contact with scientists linked to initiatives involving MARI and IITA alongside developmental implementations including water irrigation initiatives established by the Ministry of Water and Livestock Development. This confidence was brought about by the presence of strict rules and regulations which they felt governed members of the scientific community, leading them to conclude that any newly developed GM crop variety would have to be rigorously tested before its use in their farming practice.

"... even when you came to speak with us you had to go and see the District Officer [employed by the government] to get permission. If somebody wanted to grow some new crops they would need to get permission from the government and it would not be possible to test bad crops ... they would not be allowed, it would be impossible." (Respondent 1, Site C)

When asked if they would have concerns about the safety of GM crops if there were no rules or regulations regarding production of GM crop varieties in law, nine respondents reported that this would seriously affect their confidence in the safety of the crops and would possibly prevent them from becoming involved in a trial.

All of the AEOs interviewed declared that they had no significant worries with the method of GM crop variety production once it was explained to them. All three declared that they had strong faith in the government rules and regulations governing new GM crop development. Although they all assumed that these guidelines existed, they could not say whether any guidelines were in fact available and in use.

## Discussion

Our work has shown that the understanding, awareness and knowledge of GM crop technology and terminology within local farming communities in the areas of Tanzania investigated was very poor. The objective statement developed prior to data collection was used in all interviews and FGDs to facilitate further in-depth conversation. It is recognised that the use of this statement may have introduced an element of bias into the study, although the method of development of the objective statement during study design was intended to mitigate any potential issues of bias. Nevertheless, there was a willingness to gain more of an insight into biotechnology, especially regarding its potential use to improve farming practice, and in those communities where previous contact with members of the scientific community had occurred. Farmers, both male and female, had confidence in AEOs' knowledge, but we found that AEOs had limited understanding of links between agriculture, nutrition and health, and no knowledge of GM technology. These officers are required to obtain a certificate in agricultural extension following training at Agriculture Training Institutes which are under the guidance of the Ministry of Agriculture, Food and Cooperatives. This provides an excellent opportunity to raise awareness and understanding regarding GM biotechnology by provision of accurate and objective information during the training process. This would then allow these offices to provide accurate information to members of the farming community under their guidance regarding the risks and benefits of GM crops, essential to allowing these farmers to make an informed decision about the use of GM crops in their farming practice. These are important factors to consider when considering methods by which to raise awareness and improve understanding of GM crop technology.

Lack of appropriate Kiswahili terminology proved to be a noteworthy barrier to increasing basic understanding. In some cases, an English explanation was better understood by farmers. In neighbouring Kenya the Kiswahili Language Council has developed a document giving newly developed Kiswahili terminology to describe GM technology concepts and providing extended explanation of these terms [12]. In Tanzania, it would be helpful to decide whether to use English terms, and provide a full explanation of these terms, or to produce a document similar to that available in Kenya.

Farmers focused on the potential of GM crop varieties to increase overall crop production, and thus on its potential to provide improved short term food security, rather than on any potential health risks for themselves or the local ecosystem. This was in part because farmers had a high degree of confidence and trust in both government bodies and research scientists.

Self-selection of respondents could have led to only those with particular characteristics (high trust in scientists, AEOs and government officials) volunteering for interviews and FGDs. However, even if this were the case, our conclusions would remain valid, if only in relation to their impact on this homogeneous subgroup.

The results of trials of new GM crop varieties is the most significant factor which would influence farmers' decisions whether or not to use GM crop varieties in their everyday agricultural practice. Our study shows that a majority of farmers felt that they would be able to assess any comparison trial after only one growing season. This very short time frame could result in farmers misjudging the potential performance of a new GM crop variety as it requires a minimum of 3 years to assess new crop variety performance. Uncontrollable factors such as rainfall and weather conditions over a single growing season could impact on the relative performance of any new GM crop variety. It would be beneficial to raise awareness of the advantages of judging the suitability of any new crops over a longer time period and the use of a central trial site, where crop varieties that have been well established for a number of growing seasons are compared to traditional crop varieties at the same site. Central trial sites are currently used in some areas of Tanzania to assess new non-GM crop varieties: this approach could be extended to cover all farming regions, and also used if any GM crop varieties were to be trialled. Potentially worrying is farmers' openness to being influenced by incentives and their apparent inability to judge scientists' independence.

Farming communities linked agriculture and health only to the extent of trying to ensure their farming activities produced enough food to prevent them going hungry. The understanding of the health benefits which result from a diet containing essential vitamins, minerals and micronutrients was poor. Rural farming communities could significantly benefit from better understanding of the potential benefits of a more diverse diet (either by buying other foodstuffs using revenue from surplus crop sales or as a result of changes in farming practice), and from strengthening the links between agriculture, health and development. Farmers did not place value upon the potential health benefits of using vitamin- or micronutrient-fortified GM or non-GM crop varieties. Increasing the nutritional quality of a crop was much less desirable to farmers than increasing yield. Emphasis was based upon potential use of GM crops as a method to increase crop productivity to address short term food security and also a potential way to improve family finances as a result of surplus crop sales.

Farmers felt that their agricultural practices and their health could be improved by many different and varied interventions. They felt that GM crops may have the potential to improve their food production and the nutritional quality of their diets but that their development should not take precedence over other interventions including access to other labour saving technologies and improved provision and access to medical services. It is important that the scientific research community and government organisations understand this, and in response work to promote mixed and varied developments in a number of agricultural and health fields to enable the maximum possible positive outcomes to take place.

Within a global context, the results of this study support a common theme which emerges from similar research which shows that in general, understanding of GM crop technology amongst both farmers and members of the general public to be poor regardless of geographic location [13-16]. Whilst understanding remains poor, variation has been shown regarding attitudes towards the potential use of GM crops to promote increased food production and security. Within the UK and Sweden [13,14], consumers showed a low level of acceptance, raising ethical and moral concerns, while in China and Taiwan [15,16], consumers reported high acceptance towards its use in farming practice, placing its potential to improve crop production ahead of any safety concerns. The results from this study support this and suggest that in regions where food security remains a major developmental and health challenge, farmers and consumers believe the potential benefits of use of GM crop technologies outweigh any potential negative outcomes. This indicates that the willingness to use GM crops as a mechanism to improve food security is neither universal nor straightforward. This situation requires clarification, which may impact on the manner in which future development and use of GM crop technologies occurs.

## Conclusion

This study has formed a basis from which further research work in this field can be undertaken. It is important to highlight the importance of the relationship between agriculture, health and development at all levels of society in Tanzania and other developing countries in sub Saharan Africa. It is also important to continue to assess the opinions and attitudes of farmers and consumers towards the potential use of GM technologies to improve their food security and nutritional quality of their diets. Better training of AEOs in the potential advantages and disadvantages of GM biotechnology, to enable provision of better information to both farmers and non-farming members of farming communities will allow the people of Tanzania and other sub Saharan countries to make informed and autonomous decisions about whether they believe the use of GM biotechnology is an appropriate way in which to tackle issues of food security, provide improved health and drive development in their country and therefore an area where scientific development should be promoted.

## Competing interests

The authors declare that they have no competing interests.

## Authors' contributions

CPL contributed to the research proposal and application process, undertook data collection and prepared the final manuscript. JNN contributed to the research proposal and application process and edited the final manuscript. CH contributed to the research proposal and application process and edited the final manuscript. HN contributed to the research application process, undertook data collection and edited the final manuscript. All authors have seen the final edited manuscript.

## Appendix

### The prepared objective statement regarding GM crop technologies is as follows

Genetically modified crops are produced by scientists who create new forms of common crops like cassava, maize and plantain. These crops are made by a process which does not occur naturally. Scientists artificially create these crops in order to try and give them properties which mean that they may grow better or faster and be resistant to diseases or they may survive better in difficult conditions such as drought. Some examples of these GM crops are cassava plants which may survive better in drought and maize which may be resistant to the weed striga. Scientists try and do this so that more crops can be produced each year.

Many people think that these GM crops are good and should be used by farmers but many people also think that these artificial forms of normal crops should not be used.

Some people believe that using these crops which are made in an unnatural way is not safe. They believe that these crops may badly affect other crops, plants or wildlife which grow near them or may be harmful to humans who eat them once they are harvested.

On the other hand some people believe that growing these crops which may be resistant to pests, disease or drought will mean that more crops can be produced each year meaning that there is more nutritious food available for people to eat and mean that fewer people go hungry. Using these crops may also mean that farmers will be able to use less harmful chemicals which may damage the soil or wildlife and are harmful to humans. Farmers normally spray these chemicals on their crops to protect them from pests and weeds.

We are trying to speak to people to see what they think about this type of crops. It is important that you share with us what you think about this type of crops and tell us any advantages or disadvantages or questions which you may have. There are no right or wrong things to say and you should speak your mind. We are very interested in what you think and will not judge anything you say in any way.

### Semi structured interview questions used in initial interviews

Have you heard of "genetically modified crops"?

Where did you hear about them from?

What do you understand by the term genetically modified crops?

Are you aware of the use of GM foods in farming practice?

What problems do you have with your crops at the moment?

What do you think would help you to grow more and farm better?

When you have a new crop variety, who decides to choose this variety and then plant it?

Who would make the decision to grow GM crops if they were made available to you?

Would you eat GM crops?

Do you think that people would eat GM crops if you grew them?

Do you see any advantages in growing GM crops for farmers/local consumers?

Would anything stop you growing GM crops, are there other problems which are bigger or more important?

Do you have any concerns about growing GM crops?

Have you heard about GM crops from your religious leaders?

Have you heard that GM crops are bad? Where from?

Have you heard that GM crops are good? Where from?

### The Kiswahili terminology used during the discussions is as follows

#### Genetically Modified Crops

Mazao yaliyofanyiwa mabadiliko ya jeni kwa njia isiyo ya asili (Bayotechnolojia) au Mazao yaliyozalishwa kwa njia ya bayotechnolojia.

#### Genetically Modified Crops

Crops that have been created by changing/altering genes in an unnatural way (modern biotechnology) or crops that have been created by modern biotechnology.

#### Biotechnology

Bayotechnolojia, technolojia ya kisasa inayotumika kuzalisha aina mpya za mazao muhimu kama vile muhogo, mahindi, mpunga, migomba na pamba, technolojia hii huanza kufanyika maabara na huhusisha uhamishaji wa jeni zilizokusudiwa kutoka katika katika mmea au kiumbe hai fulani na kuweka katika mmea mwingine ili kupata matokeo fulani. Kama vile mmea kuwa na ukinzani au kustahimili magonjwa, wadudu wa haribifu au ukame, mmea kukua kwa ubora na kwa haraka zaidi na mmea kuzaa zaidi.

#### Biotechnology

Modern technology which is used to create new varieties of important crops like cassava, maize, rice, banana and cotton, this technology usually starts in the laboratory and it involves the transfer or altering of specific genes from a crop plant (or living thing) to another crop plant of the same or different species in order to get a crop with desired characteristics. Examples are crops which are resistant or tolerant to drought, pests and diseases, crops which grow well, with short growing period and with high yield.

#### Gene

Jeni, sehemu ya kinasaba inayorithisha tabia au umbile fulani katika kiumbe hai (wanyama na mimea).

#### Gene

A part of DNA in a cell which controls the inheritance of a certain characteristic or physical form of a living organism.

#### DNA

Kinasaba, kemikali katika chembe za uhai za mmea au mnyama ambazo husimamia muundo na kazi za kila chembe za uhai na huchukua taarifa za kijenetiki wakati wa kuzaliana kwa mimea au wanyama.

#### DNA

Deoxyribonucleic acid, the chemical at the centre of the cells of living things, which controls the structure and purpose of each cell and carries the genetic information during reproduction.

## Pre-publication history

The pre-publication history for this paper can be accessed here:

http://www.biomedcentral.com/1471-2458/10/407/prepub
